# Modulation of the unfolded protein response by the human hepatitis B virus

**DOI:** 10.3389/fmicb.2014.00433

**Published:** 2014-08-19

**Authors:** Catalin Lazar, Mihaela Uta, Norica Branza-Nichita

**Affiliations:** Department of Viral Glycoproteins, Institute of Biochemistry of the Romanian AcademyBucharest, Romania

**Keywords:** hepatic viruses, ER stress, degradation, autophagy

## Abstract

During productive viral infection the host cell is confronted with synthesis of a vast amount of viral proteins which must be folded, quality controlled, assembled and secreted, perturbing the normal function of the endoplasmic reticulum (ER). To counteract the ER stress, cells activate specific signaling pathways, designated as the unfolded proteins response (UPR), which essentially increase their folding capacity, arrest protein translation, and degrade the excess of misfolded proteins. This cellular defense mechanism may, in turn, affect significantly the virus life-cycle. This review highlights the current understanding of the mechanisms of the ER stress activation by Human Hepatitis B virus (HBV), a deadly pathogen affecting more than 350 million people worldwide. Further discussion addresses the latest discoveries regarding the adaptive strategies developed by HBV to manipulate the UPR for its own benefits, the controversies in the field and future perspectives.

## Introduction

An important amount of experimental data accumulated in the last decade have not only demonstrated that viruses can induce endoplasmic reticulum (ER) stress in mammalian cells, but importantly, that the cellular response to this stress may play a crucial part in the evolution of the disease (Netherton et al., 2004; Tardif et al., 2005). This is not surprising, since an infected cell must manage a vast quantity of viral proteins that are synthesized over a short period of time during productive infection, often leading to perturbation of the ER homeostasis, protein misfolding and aggregation.

Discovered more than 40 years ago, with an efficient vaccine developed against it, the Human Hepatitis B virus (HBV) infection is still a frequent viral disease and a major cause of chronic liver pathogenesis. About 350 million people are currently HBV carriers worldwide and at high risk to develop hepatocellular carcinoma (HCC), the third leading cause of cancer death in humans. Despite decades of intensive research and comprehensive investigations at large scale, such as by using proteomics and transcriptomics technologies, the HBV interactions with the host cells and the molecular mechanisms underlying the viral pathogenesis and progression are not clearly understood. The lack of an efficient and robust *in vitro* infectivity model has been a major drawback, preventing in depth studies in a natural infection system (Gripon et al., 2002). However, HBV is one of the few viruses demonstrated to induce ER stress, with strong support by *in vivo* data.

**Figure 1:**
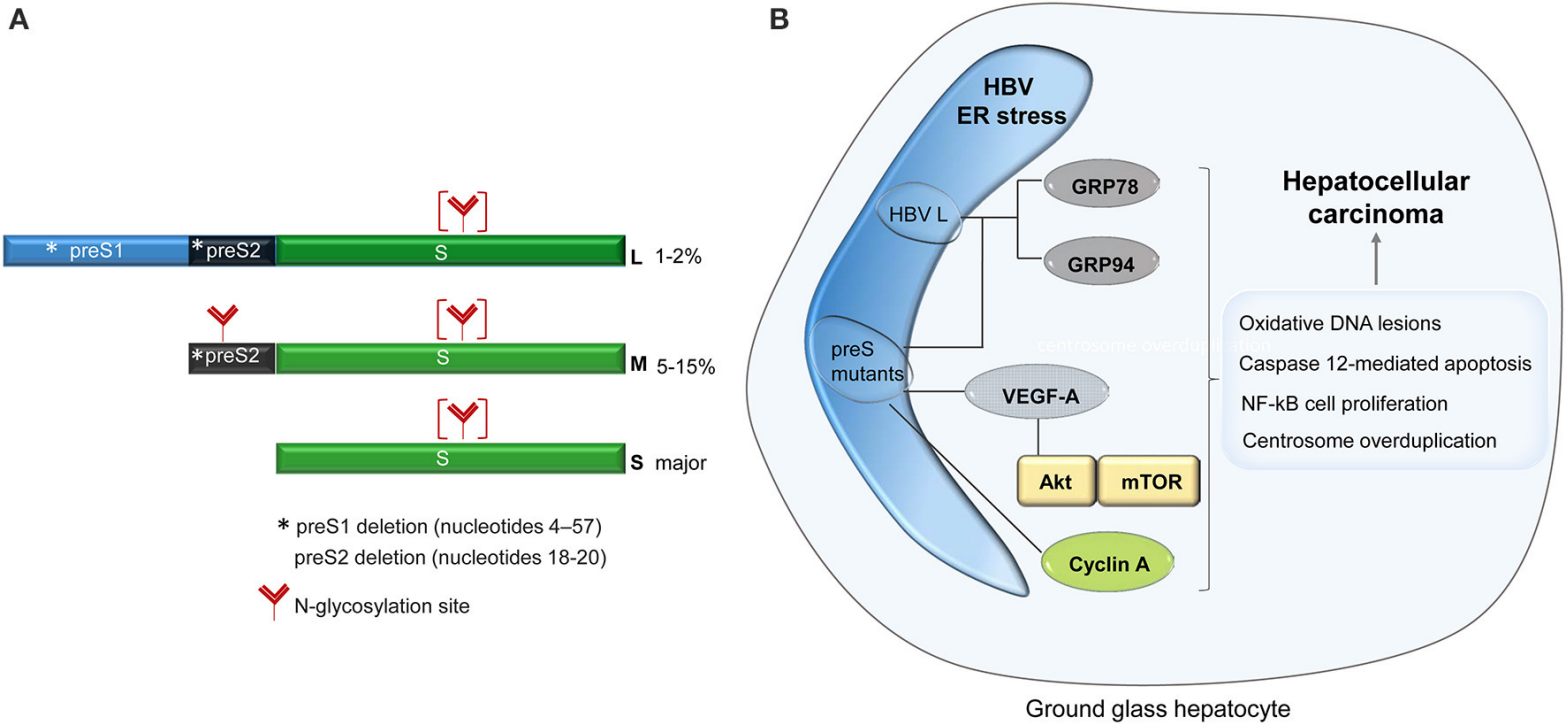
**Schematic representation of the HBV surface proteins**. The asterisk shows the location of the major mutations identified in patients with ground glass hepatocytes **(A)**. Signaling pathways activated in ground glass hepatocytes, with potential role in carcinogenesis **(B)**.

HBV is the representative of the *Hepadnaviridae* family of DNA viruses. Its genome consists of a partially double-stranded DNA molecule of 3.2 kb encoding the envelope (surface) proteins, a core protein, the reverse transcriptase and the regulatory X protein (HBx), within four overlapping open reading frames (ORF) (Schadler and Hildt, 2009). The surface proteins, namely the large (L), middle (M), and small (S) are synthesized from three different initiation codons within the same ORF and share the tetra-spanning transmembrane S domain. In addition to S, the M and L proteins contain the preS2 and the preS1-preS2 regions, respectively at their N-terminal end (Figure 1A). The surface proteins are co-translationally inserted into the ER membrane, fold and oligomerize by extensive disulfide bonding, before being transported to the budding sites for assembly into virions and non-infectious subviral particles (SVP) (Chai et al., 2008). The preS1 domain adopts a dual topology at the ER membrane enabling the interaction of the L protein with both, the core protein in the cytoplasm and the viral receptors at the plasma membrane (Lambert and Prange, 2001). Due to this intriguing feature, the L protein is indispensable to HBV assembly and entry into hepatocytes, but not to SVP formation. Interestingly, when expressed alone, the L protein assembles into particles that are retained within the ER lumen. Secretion of the L protein is rescued when either M and/or S proteins are co-expressed and incorporated into these particles (Bruss and Ganem, 1991). Similarly, excessive production of L, over M and S, results in retention of all three proteins and oligomers as well as virions, demonstrating that the ratio between the surface proteins plays a crucial role in the HBV life-cycle (Chisari et al., 1986). Therefore, it is not surprising that expression of the surface proteins is tightly regulated by different promoters, namely, the preS1 promoter, which controls the L transcript and the S promoter regulating transcription of the M and S mRNAs. This control results in differential expression of the surface proteins, S being the most abundant of them, while M and L are expressed at much lower levels of about 5–15% and 1–2%, respectively (Yokosuka and Arai, 2006).

## HBV induces ER stress

The first indication of the ER stress and the intracellular morphological modification induced by HBV infection came more than 40 years ago, from studies of cirrhotic and carcinomas liver biopsies, showing a strong hypertrophy of the ER within hepatocytes. Initially associated with a disregulated glycogen metabolism, the presence of the altered subcellular structures was later clearly related to the expression of HBV surface proteins (Hadziyannis et al., 1973). Due to their microscopic appearance, these cells were termed “ground glass hepatocyte” (GGH) and their detection became an important parameter in the diagnostic of HBV-related pathogenesis (Pópper, 1975). The phenotype was classified in type I, characterized by a random distribution of the GGH in the hepatic tissue, and type II, where larger regions of clustered GGH are observed, occurring usually at later stages of infection (Fan et al., 2000, 2001). A potential mechanism of this unusual intracellular accumulation of the secretory proteins was suggested with the discovery that mutations in the pre-S1 domain of the L protein inhibited secretion of the surface proteins, inducing the GGH phenotype (Xu and Yen, 1996). Moreover, a deletion naturally occurring in the pre-S2 region of the same protein produced similar effects, suggesting that mutations in the entire pre-S region are responsible for the accumulation of the viral proteins and the development of the GGH morphology (Fan et al., 2000, 2001). The level of this accumulation appears to play a crucial role in triggering the ER stress since natural pre-S2 deletion leading to a moderate retention of the HBV envelope proteins were not able to induce cellular toxicity, at least *in vitro* (Tai et al., 2002).

The ability of secretion-incompetent pre-S mutants to specifically activate ER stress signaling pathways in host cells was further demonstrated by the up-regulation of well-established stress sensors such as GRP78 (BiP), GRP94, and ER-resident kinases (Wang et al., 2003). These stress signals could also induce oxidative DNA lesions and mutagenesis (Chen et al., 2006; Hsieh et al., 2007), caspase 12-mediated apoptosis, or NF-kB-mediated cell proliferation with an important role in cancer development (Qu et al., 2004). Interestingly, the Akt/mTOR pathway and the VEGF-A synthesis were found significantly activated in GGHs (Yang et al., 2009); it is therefore tempting to speculate that the ER stress induced by the pre-S mutants plays a key role in progression of HBV pathogenesis, leading to neoplastic lesions of the liver in chronically infected patients. The observation that the cell-cycle progression is directly affected by the ER stress provides additional data in support of this hypothesis. The ER accumulation of a pre-S2 mutant protein promotes cyclin A cleavage by the calcium-dependent protease μ-calpain, followed by its translocation from the nucleus into the cytoplasm and centrosome overduplication (Wang et al., 2012). The cytoplasmic distribution of cyclin A reported in transgenic mouse livers expressing pre-S2 mutants appears to favor this mechanism (Wang et al., 2005) (Figure 1B). However, these results must be interpreted with care, as overexpression of the wild-type L protein in transgenic mice was shown to be sufficient to induce aberrant changes in the hepatocyte, “ground-glass” appearance, cell death and nodular hyperplasia of the liver (Chisari et al., 1987).

Interestingly, a point L77R mutation occurring naturally in the cytosolic loop of the S protein results in retention of the S protein in the ER-Golgi compartment, probably due to impaired folding (Chua et al., 2005). Despite of this significant accumulation, no ER stress was detected in cells expressing this mutant variant, raising the question whether the pre-S-independent accumulation of the envelope proteins is able to induce UPR signaling.

## Regulatory mechanisms activated in HBV-infected cells to reduce the ER stress

To alleviate the ER stress, cells initiate a complex signaling cascade termed the unfolded protein response (UPR), which relies on activation of three complex signaling pathways at the ER level and continued in the cell nucleus. The sensors of the ER stress and the key regulators of these pathways are the transmembrane proteins inositol-requiring protein 1 (IRE1) α and β, the activating transcription factor 6 (ATF6) α and β and the protein kinase RNA-like ER kinase (PERK), all regulated by BiP. Binding of these sensors to BiP maintains them in an inactive state; this process is competed by the accumulation of unfolded proteins within the ER, which also require interaction with BiP to prevent terminal misfolding and aggregation. Eventually BiP is released from the interaction with IRE1, ATF6, and PERK (Kimata et al., 2004) which promotes their activation by dimerization and autophosphorylation (PERK and IRE1) (Liu et al., 2000; Su et al., 2008) or by trafficking to the Golgi apparatus and further proteolytic processing (ATF6) (Ye et al., 2000; Chen et al., 2002). Activated IRE1 directs splicing of the full-length (unspliced) XBP1 mRNA (XBP1u) generating a shorter variant. This encodes a highly active transcription factor (XBP1s) which is able to induce transcription of selected genes by binding the ER stress response elements (ERSE) containing the consensus sequence CCAAT-N9-CCACG (Yoshida et al., 1998, 2001; Lee et al., 2002). Activated PERK phosphorylates the α subunit of the eukaryotic initiation factor-2 α (eIF2α), blocking the assembly of the ribosomal complex and thus protein translation (Ma et al., 2002). ATF6 cleavage within the Golgi releases its N-terminal domain which translocates to the nucleus and functions as a transcription factor on target genes, including that encoding for XBP1 (Haze et al., 1999). Collectively, these complex transcriptional activities and cross-talks between the signaling pathways result in: (a) attenuated protein translation (b) increased transcription of ER chaperone genes; (c) up-regulation of key molecules of the ER degradation machinery.

Interestingly, a first regulatory mechanism involved in alleviation of the ER stress produced by HBV infection was first described at viral level. It was observed that overexpression of the L protein increased the transcriptional activity of the S promoter by up to 10 folds, while neither M, S, or a secreted variant of L had a similar effect (Xu et al., 1997). Moreover, L accumulation also activated the promoters of the genes encoding for GRP78 and GRP94, the two chaperone proteins up-regulated during ER stress (Ramakrishnan et al., 1995; Roy and Lee, 1995). Activation of these promoters by the L protein was shown to depend on the transcription nuclear factor NF-Y and its binding to the CCAAT element, also present in the S promoter. Two *cis*-acting elements in the S promoter, namely Z1 and Z2 flanking the CCAAT region were also required for its activation by the ER stress, through a, yet unknown, transcription factor called “Z.” The activation of the S promoter was later shown to be independent of the PERK pathways (Huang et al., 2005); rather, expression of the “Z” factor was induced by XBP1(s) the transcription factor resulted following IRE1 activation (Calfon et al., 2002). Since XBP1(s) and ATF6-alpha interact with each other to form heterodimers and bind to similar DNA sequences (Lee et al., 2002), a potential involvement of the ATF6-alpha pathway in this regulation, although not directly demonstrated, could not be excluded in this study.

An interesting hypothesis emerging from this investigation was based on the observation that induction of the “Z” factor by the ER stress was restricted to certain cell types, including liver and kidney cells, but not fibroblasts. Given the ubiquity of the UPR signaling pathways, this result is rather unusual and may indicate that the cells have also evolved specific mechanisms in response to ER stress stimuli that are characteristic to a specific tissue.

HBx and S proteins were also shown to activate the IRE1/XBP1 branch of the UPR, in independent studies. The HBx protein expressed transiently in Hep3B and HepG2 cells resulted in a significant increase of the XBP1 promoter activity, by up to 7 folds, as demonstrated using a luciferase expression reporter (Li et al., 2007). Moreover, splicing of the Xbp-1 mRNA occurred efficiently in these cells, and the presence of XBP1(s) was clearly evidenced in the same study. Splicing of the Xbp-1 mRNA was also shown in HepG2.2.2.15 cells, which contain two copies of the HBV genome and support HBV replication, assembly and secretion of SVPs and of fully infectious virions. The process depended on the HBx expression level, further supporting the involvement of this protein in UPR activation (Li et al., 2007). Similarly, Huh7 hepatoma cells overexpressing the S protein contained both, the precursor and the spliced form of the Xbp-1 mRNA, suggesting that the envelope protein is also able to trigger UPR via the IRE1/XBP1 pathway (Li et al., 2011). However, whether or not XBP1-specific target genes were actually activated in these systems had not been investigated in either study.

## Activation of the ER-associated degradation (ERAD) by HBV

The first UPR target demonstrated to depend entirely on the IRE1-XBP1 pathway was the gene encoding for a member of the ER degradation-enhancing, mannosidase-like family of proteins (EDEM1) (Yoshida et al., 2003). EDEM1 and its two homologs EDEM2 (Mast et al., 2005) and EDEM3 (Hirao et al., 2006) belong to the glycoside hydrolase 47 family and are believed to play an important role in alleviating the ER stress during UPR, by targeting misfolded glycoproteins to ERAD (Olivari and Molinari, 2007).

Our investigation on the ERAD function in the HBV life-cycle revealed strong evidence that HBV and the IRE1-XBP1 branch of the UPR are in tight, mutual relationship. Synthesis of the transcripts encoding for the three members of the EDEM family was significantly increased in HepG2.2.215 cells hosting stable HBV replication, or Huh7 cells replicating the virus in a transient manner (Lazar et al., 2012). Moreover, while HepG2 cells express only trace amounts of EDEM1, expression of this protein became clearly detectable in HepG2.2.215 cells. It is important to note that activation of EDEM synthesis was not related to HBV replication and nucleocapsids accumulation within cells, as it occurred with similar efficiency in the presence of lamivudine, a strong DNA replication inhibitor (Doong et al., 1991). Rather, overexpression of the surface proteins appeared sufficient to induce EDEM up-regulation, which is in agreement with the ability of these proteins to activate the IRE1/XBP1 branch of the UPR (Li et al., 2011). EDEM1 overproduction induced a significant degradation of the wild-type S, M, and L proteins, when expressed either independently, or in the context of a full replication-cycle. This observation was surprising, since EDEM has been traditionally involved in disposal of misfolded proteins and therefore, it called for a deeper investigation.

The incapacity of the ER folding machinery to deal with an excess of viral protein synthesis could be a possible explanation for this result. However, silencing of the endogenous EDEM1 resulted in intracellular accumulation of the HBV surface proteins, accompanied by a significant increase of their secretion. This strongly suggests that an important fraction of folding-, assembly-, and secretion-competent envelope proteins is delivered to degradation during HBV protein synthesis. Interestingly, the proteins rescued from degradation in EDEM1 knocked-down cells were recruited for nucleocapsid envelopment, increasing the amount of secreted virions. Thus, despite the availability of correctly folded surface proteins, their trafficking to the corresponding virus particles and SVP budding sites appear to be competing, rather than successive processes.

**Figure 2:**
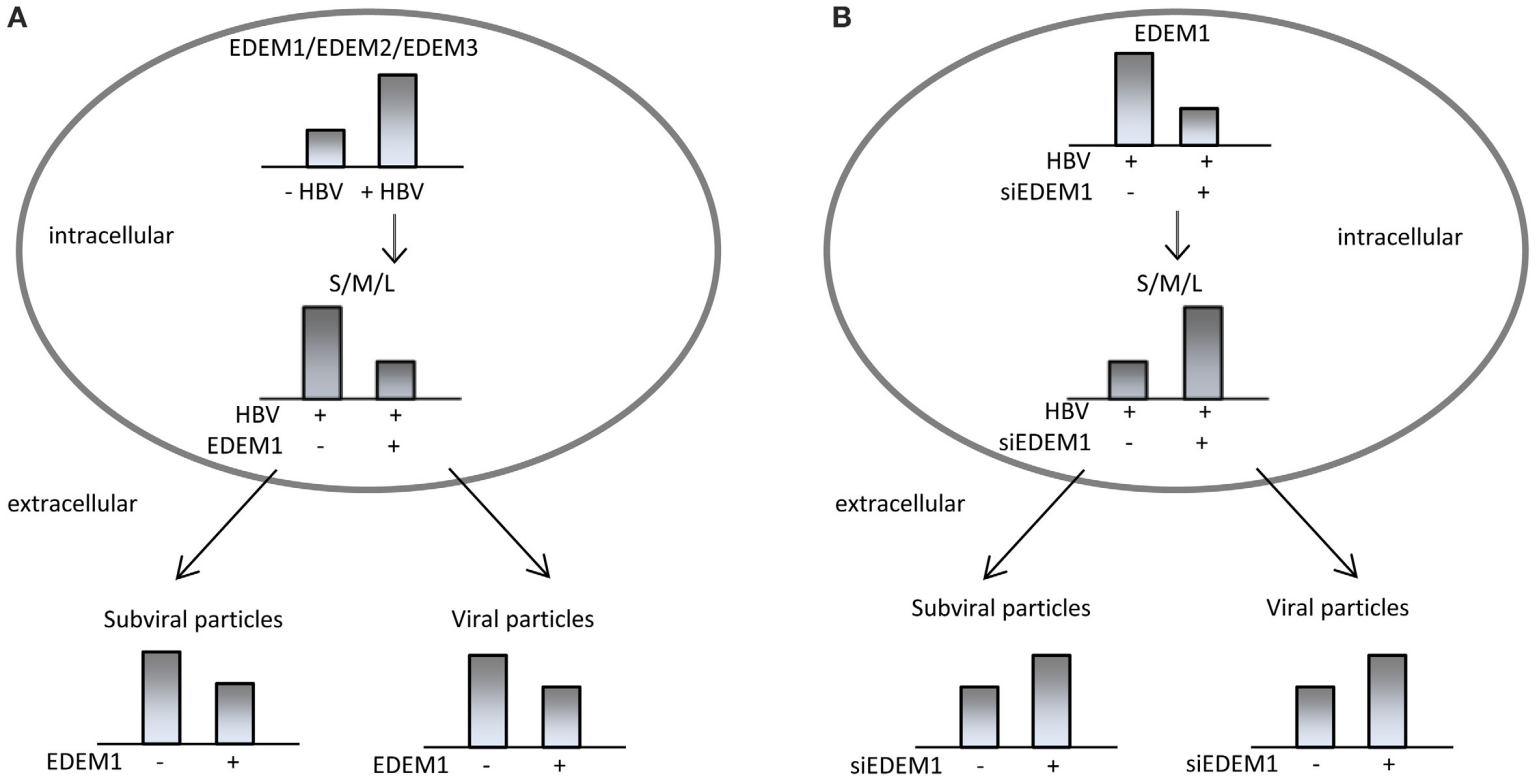
**HBV interaction with the ER-associated degradation pathway**. HBV activates expression of EDEMs, which promote degradation of wild-type, assembly-competent HBV surface proteins, reducing the production of subviral and viral particles **(A)**. Silencing of endogenous EDEM1 in HBV replicating cells induces accumulation of folding-competent envelope proteins and increases secretion of subviral and viral particles **(B)**.

In search for a mechanism of action underlying the effects of EDEM1 modulation on the HBV life-cycle, a direct interaction between the endogenous protein and the viral polypeptides was evidenced by co-immunoprecipitation. EDEM1 co-precipitated both, the glycosylated and non-glycosylated isoforms of the envelope proteins. Moreover, investigation of the surface proteins oligomerization in EDEM1-depleted cells showed that EDEM1 acts early during surface protein synthesis, most probably before their assembly into oligomers. Collectively, these experiments point to an N-glycan-independent mechanism of EDEM1 binding to the viral substrates, possibly involving the common S-domain. These data are in support of a more recent model of EDEM1-substrate recognition, implying a glycan-independent binding of the ERAD targets (Cormier et al., 2009). The evidence of EDEM1 interaction with wild-type proteins and the observation that glycoprotein substrates are still targeted to ERAD in the absence of mannose trimming, when EDEM1 is up-regulated (Ron et al., 2011), have added new angles to this model. It was proposed that the significant induction of EDEM1 expression in HBV-replicating cells promotes extraction of conformation-viable viral polypeptides from the ER-resident protein quality control cycle and their premature delivery to degradation (Lazar et al., 2012). This has important consequences on the HBV life-cycle (summarized in Figure 2), resulting in reduction of SVP and virion production.

The concept that wild-type proteins can also be degraded during UPR is supported by data obtained from studies on Human Hepatitis C virus (HCV), showing a direct involvement of EDEM1 in regulating the production of wild-type envelope proteins at post-translational level (Saeed et al., 2011). EDEM1 overexpression promotes degradation of the E1 and E2 surface proteins, which results in down-regulation of HCV particles production. Conversely, the E2 protein shows greater stability in EDEM1 knocked-down cells, which also produce an increased amount of infectious HCV, with no effect on viral replication (Saeed et al., 2011).

An important question arising from these investigations regards the validity of this concept for the UPR induced during conditions other than viral infections. Most of the pioneering works leading to characterization of the UPR signaling pathways have used model proteins undergoing severe conformational changes, due to genetic mutations or other environmental factors, often leading to pathogenesis. These conditions are known as “folding diseases,” of which some neurodegenerative disorders, diabetes, obstructive pulmonary disease are best documented (Yoshida, 2007). It would be interesting to extend this research to other wild-type cellular proteins to understand the subtleties of the molecular discrimination between correctly folded and misfolded proteins and their degradation and identify the cellular factors involved in this recognition.

Unlike the case of “folding diseases,” the amount of data regarding the UPR in viral infections is relatively limited and controversial; in addition to HBV and HCV discussed above, only a few other viruses, such as Borna Disease Virus (Williams and Lipkin, 2006), murine leukemia virus (Dimcheff et al., 2006), rotaviruses (Trujillo-Alonso et al., 2011), or the West Nile virus (Ambrose and Mackenzie, 2011) have been reported to induce ER stress. Most of these viruses have adapted mechanisms to use the UPR signaling pathways to their own benefit, whether this is assistance for proper protein folding or replication. In the case of the HBV/HCV, it is tempting to speculate that activation of the ERAD pathway would limit the amount of surface proteins available for nucleocapsid envelopment, on one hand, and avoid extensive damage of the ER due to viral protein accumulation, on the other hand, thus enabling the evolution of infection toward chronicity.

## HBV induces autophagy

**Figure 3:**
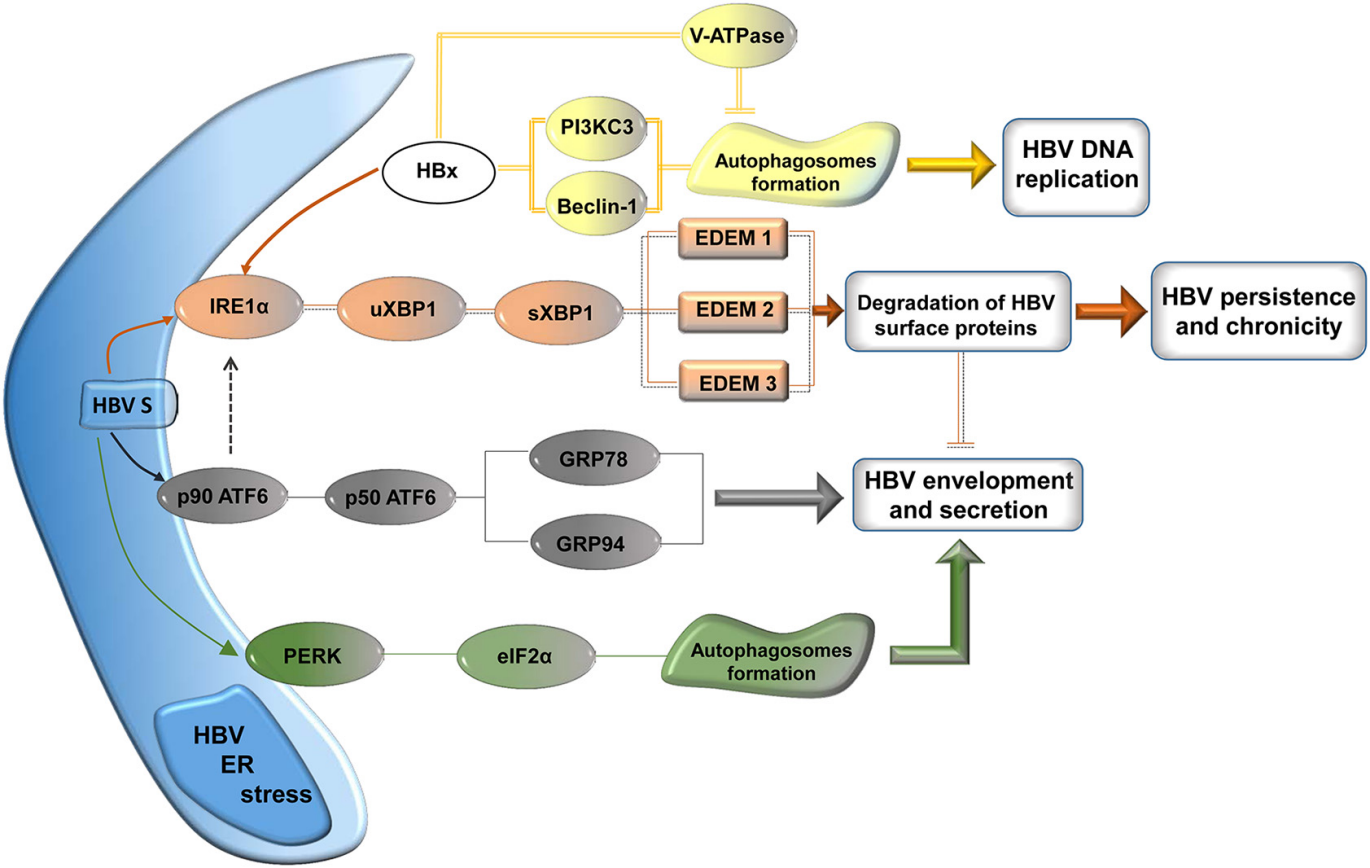
**Signaling pathways modulated by HBV for its own benefit**. The surface proteins activate the UPR leading to autophagy, involved in nucleocapsid envelopment and virus particle secretion. Activation of the IRE1 pathways results in up-regulation of the EDEM family of proteins, which reduces the load of viral surface proteins, possibly contributing to HBV persistence and chronicity. HBx induces autophagosome formation via PI3KC3/Beclin-1 activation, which is involved in HBV DNA replication. Transport of the V-ATPase to autophagosomes is possibly impaired by HBx, resulting in elevated vacuolar pH and lack of protein degradation.

It has been established that prolonged UPR eventually leads to cells death by apoptosis. However, before making the ultimate choice, cells under severe ER stress are able to recruit survival pathways, such as autophagy. Autophagy is a catabolic process, highly conserved during evolution, involving degradation of long-lived macromolecules and defective organelles within double membrane compartments, called autophagosomes (He and Klionsky, 2009). Interestingly, several viruses, including HBV, were shown to induce autophagy and further exploit it in productive or unproductive infections (Pratt and Sugden, 2012). Activation of autophagy by HBV has been clearly demonstrated by two independent groups (Sir et al., 2010; Li et al., 2011), leading to one of the most interesting, recent controversy in the HBV field (Figure 3). Both groups have observed conversion of the autophagic marker LC3 from the cytosolic to the lipidated, autophagosome-associated form in HBV transfected cells (Sir et al., 2010; Li et al., 2011) and livers of HBV-infected patients or transgenic mice (Sir et al., 2010). Autophagosomes formation in the presence of HBV was also convincingly confirmed by electron and confocal microscopy; notably, this was not accompanied by protein degradation, suggesting a strong interference of HBV with the autophagy signaling pathway preventing further clearance of the engulfed macromolecules (Sir et al., 2010; Li et al., 2011). However the two studies diverge in their proposed mechanisms of HBV-induced autophagy and the interpretation of the role played by this process at different steps of the viral life-cycle. A first indication of a viral factor involvement in autophagy induction was reported by Tang and collaborators (Tang et al., 2009). It was shown that Beclin-1 expression in hepatic and hepatoma cells was modulated by HBx, which acts at transcriptional level, activating its promoter. This induces autophagy under nutrient starvation conditions, which could be inhibited by Beclin-1 silencing with specific siRNA. Intriguingly, Beclin-1 up-regulation was not confirmed in a subsequent study, despite HBx being also indicated as the major factor triggering autophagy (Sir et al., 2010). Rather, the process appeared to be mediated by activation of PI3KC3, an enzyme playing a critical role autophagy initiation, following a direct interaction with HBx.

Very recently, an independent study provided additional evidence in support of the HBx role in the formation of autophagosomes and a potential mechanism for the absence of protein degradation observed, despite the activated autophagy (Liu et al., 2014). It was shown that lysosomal activity was significantly perturbed in HBx-expressing cells, possibly due to mistrafficking of the V-ATPase involved in lysosomes acidification. This results in accumulation of defective lysosomes containing immature hydrolases, such as Cathepsin D, which are unable to degrade the cargo proteins (Liu et al., 2014).

However, another series of experiments have implied that S protein expression is sufficient to activate autophagy by a mechanism involving cellular stress, excluding a potential contribution of HBx (Li et al., 2011). High amounts of S protein expressed either alone or in the context of the full viral replication resulted in phosphorylation of PERK and eIF2α, its direct effector. Similarly, XBP1 mRNA splicing was induced by S, while only the unspliced form could be detected in control cells, in the absence of the viral protein. ATF6 activation was also investigated in these cells; although the cleavage products could not be directly evidenced, up-regulation of ATF6 downstream effectors, such as GRP94 (Eletto et al., 2010) was unambiguously demonstrated. Collectively, these data suggest that accumulation of the S protein activates the ER stress pathways regulated by PERK, ATF6, and IRE1. Moreover, knock-down of either signaling route efficiently prevented LC3 lipidation and autophagosome formation induced by the HBV surface protein, further supporting the hypothesis above. Finally, an important fraction of the S protein associated with the autophagosome membrane and a direct interaction with LC3 was demonstrated, providing a potential mechanism for the effects of the HBV-induced autophagy on the viral life-cycle (Li et al., 2011).

These effects have raised additional controversy between the two groups. One group suggested that autophagy is critical for the viral DNA replication, while little effects were observed on the RNA synthesis or its packaging into nucleocapsids (Sir et al., 2010). Based on several lines of evidence, including: (a) the partial co-localization of the HBV core and surface proteins with LC3, (b) cell treatment with 3-MA, a PI3KC3 inhibitor, (c) specific down-regulation of Vps34, the catalytic subunit of PI3KC3 and of Atg7, an enzyme involved in autophagosomes formation, it was proposed that early autophagic vacuoles may function as platforms for viral DNA replication and assembly. Alternatively, an indirect role of the autophagosomes in HBV replication, by hosting signaling molecules involved in regulation of the core protein phosphorylation is also likely. Important evidence for a role of autophagy in the production HBV particles *in vivo* was provided by the experiments using HBV transgenic mice with liver-specific knockout of another autophagy initiating factor, Atg5 (Tian et al., 2011). The impaired autophagy in this system resulted in a significant reduction of both, HBV DNA and SVP secretion in the mice sera.

In contrast, the second group suggested that the UPR- induced autophagy is required for efficient envelopment of the HBV nucleocapsids, while the DNA replication is only moderately affected. This conclusion was based on (a) the decreased secretion of enveloped virions from cells treated with 3-MA, (b) the increased amount of extracellular enveloped virions in the presence of autophagy inducers, such as rapamycin and starvation.

The lines of evidence provided by the two groups in support of their conclusions appear compelling and difficult to reconcile at a first glance. However, it is very likely that in fact, the two mechanisms operate together, one or the other prevailing according to the amount of viral protein expressed, or the hepatoma cell line used, which may activate different levels of ER stress. It is important to note, for instance, that the first study focused on DNA replication in autophagy-inhibited cells, while the efficiency of virion envelopment and secretion were not assessed (Sir et al., 2010). Similarly, a moderate effect of the UPR-induced autophagy on DNA replication was also observed in the second study, which analyzed in more detail virion assembly and secretion (Li et al., 2011). It would be interesting for future investigations to address the relationship between the HBx and the envelope proteins in the HBV-induced autophagy, in a more systematic manner and in the context of a complete viral life-cycle, ideally in a natural infection system.

Activation of the UPR and autophagy signaling during mild ER stress clearly favors the recovery of the cellular homeostasis. As mentioned above, prolonged ER stress may trigger apoptosis to remove the irreversibly damaged cells. This is a complex process which relies on activation of transcription factors (e.g., the C/EPB homologous protein -CHOP), phosphatases and kinases (e.g., the protein phosphatase 1—PP1, the apoptosis signal-regulating kinase 1—ASK1, and JNK) (Nishitoh, 2012), which, in turn, regulate downstream pro-apoptotic factors (Wei et al., 2001). The ability of HBV to induce apoptosis is a matter of intense debate, which is far from being concluded. One set of experimental data indicates the absence of apoptosis in HBV replicating cells (Schulze-Bergkamen et al., 2003) or inhibition of apoptosis by HBV, by several mechanisms (Huo et al., 2001; Marusawa et al., 2003; Liu et al., 2013). In addition, it was recently shown that artificially-induced apoptosis is detrimental to the HBV life-cycle (Arzberger et al., 2010). In contrast, other reports suggest that overexpression of the HBx protein can trigger apoptosis of liver cells, in a p53-dependent (Wang et al., 2008) or independent manner (Terradillos et al., 1998). However, it remains to be established whether expression of the HBx protein can reach this critical levels in a natural infection and how the stage and progression of infection may influence the pro- or anti-apoptotic responses observed in different HBV experimental systems.

## Conclusion remarks

While the HBV-induced ER stress and UPR signaling have been clearly established, the consequences of this activation on both, the host cell and the virus life-cycle, are far from being elucidated. There are many interesting issues that deserve deeper scrutiny, such as the relationship between the ER stress and carcinogenesis and the UPR modulation by HBV for own benefits, whether this is protein folding, genome replication, virion assembly, or establishment of chronic infection (Figure 3). It is important to note that studies regarding the UPR at early stages of HBV infection have not even been addressed. This may be explained by the difficulty to investigate HBV infection *in vitro* using the infectivity models available. It is expected that the recent discoveries of cellular factors facilitating HBV entry and the development of new, improved cellular systems, permissive for HBV infection (Yang et al., 2014), will help expand this research by approaching the current controversies in the context of the whole viral infection.

### Conflict of interest statement

The authors declare that the research was conducted in the absence of any commercial or financial relationships that could be construed as a potential conflict of interest.

## Abbreviations

Akt: protein kinase B
Atg: autophagy-related
BiP: immunoglobulin heavy chain binding protein
GGH: ground glass hepatocytes
GRP78: glucose-regulated protein of 78 kDa
GRP94: glucose-regulated protein of 94 kDa
LC3: microtubule-associated light chain 3
3-MA: 3-methyladenine
mTOR: mammalian target of rapamycin
NF-kB: nuclear factor kB
PI3KC3: class III phosphatidylinositol 3-kinase
V-ATPase: vacuolar ATPase
VEGF-A: vascular endothelial growth factor A.
